# Laboratory and clinical management capacity for invasive fungal infections: the Italian landscape

**DOI:** 10.1007/s15010-023-02084-x

**Published:** 2023-09-01

**Authors:** Antonio Vena, Matteo Bassetti, Laura Mezzogori, Francesco Marchesi, Martin Hoenigl, Daniele Roberto Giacobbe, Silvia Corcione, Michele Bartoletti, Jannik Stemler, Livio Pagano, Oliver A. Cornely, Jon Salmanton-García

**Affiliations:** 1 Department of Health Sciences (DISSAL), University of Genoa, Genoa, Italy; 2 Clinica Malattie Infettive, IRCCS Ospedale Policlinico San Martino, Genoa, Italy; 3 IRCCS Regina Elena National Cancer Institute, Rome, Italy; 4 Division of Infectious Diseases, Excellence Center for Medical Mycology, Department of Internal Medicine, Medical University of Graz, Graz, Austria; 5 Division of Infectious Diseases, ECMM Center of Excellence for Medical Mycology, Medical University of Graz, Graz, Austria; 6 BioTechMed, Graz, Austria; 7 Department of Medical Sciences, Infectious Diseases, University of Turin, Turin, Italy; 8 Tufts University School of Medicine, Boston, MA USA; 9 Department of Biomedical Sciences, Humanitas University, Milan, Italy; 10 Infectious Disease Unit, IRCCS Humanitas Research Hospital, Rozzano, Milan Italy; 11 Faculty of Medicine, Institute of Translational Research, Cologne Excellence Cluster On Cellular Stress Responses in Aging-Associated Diseases (CECAD), University of Cologne, University Hospital Cologne, Cologne, Germany; 12 Department I of Internal Medicine, Center for Integrated Oncology Aachen Bonn Cologne Duesseldorf (CIO ABCD) and Excellence Center for Medical Mycology, University of Cologne, Herderstraße 52-54, 50931 Cologne, Germany; 13 Partner Site Bonn-Cologne, German Centre for Infection Research (DZIF), Cologne, Germany; 14 Hematology Unit, Fondazione Policlinico Universitario Agostino Gemelli, IRCCS, Rome, Italy; 15 Hematology Unit, Università Cattolica del Sacro Cuore, Rome, Italy; 16 Faculty of Medicine, Center for Molecular Medicine Cologne (CMMC), University of Cologne, University Hospital Cologne, Cologne, Germany; 17 Infectious Diseases Unit, IRCCS for Oncology and Neuroscience, San Martino Policlinico Hospital, Largo Rosanna Benzi, 10, 16132 Genoa, Italy; 18 Faculty of Medicine, University of Cologne, University Hospital Cologne, Clinical Trials Centre Cologne (ZKS Köln), Cologne, Germany

**Keywords:** Italy, Mycology, Therapeutic drug monitoring, Antifungals, Diagnostic capacity, microscopy, Culture, serology, Antigen, Molecular test

## Abstract

**Background:**

We assessed the laboratory diagnosis and treatment of invasive fungal disease (IFD) in Italy to detect limitations and potential for improvement.

**Methods:**

The survey was available online at www.clinicalsurveys.net/uc/IFI management capacity/, and collected variables such as (a) institution profile, (b) perceptions of IFD in the respective institution, (c) microscopy, (d) culture and fungal identification, (e) serology, (f) antigen detection, (g) molecular tests, (h) susceptibility testing and (i) therapeutic drug monitoring (TDM).

**Results:**

The laboratory capacity study received responses from 49 Italian centres, with an equitable geographical distribution of locations. The majority of respondents (*n* = 36, 73%) assessed the occurrence of IFD as moderate-high, with *Aspergillus* spp. being the pathogen of highest concern, followed by *Candida* spp. and Mucorales. Although 46 (94%) of the institutions had access to microscopy, less than half of them performed direct microscopy on clinical specimens always when IFD was suspected. Cultures were available in all assessed laboratories, while molecular testing and serology were available in 41 (83%), each. Antigen detection tests and antifungal drugs were also generally accessible (> 90%) among the participating institutions. Nevertheless, access to TDM was limited (*n* = 31, 63%), with a significant association established between therapeutic drug monitoring availability and higher gross domestic product per capita.

**Conclusions:**

Apart from TDM, Italy is adequately prepared for the diagnosis and treatment of IFD, with no significant disparities depending on gross domestic product. Future efforts may need to focus on enhancing the availability and application of direct microscopic methods, as well as TDM, to promote optimal treatment and better patient outcomes.

**Supplementary Information:**

The online version contains supplementary material available at 10.1007/s15010-023-02084-x.

## Introduction

Invasive fungal diseases (IFDs) are a significant clinical threat, affecting a wide spectrum of patients, notably those who are immunocompromised or undergoing invasive procedures due to their associated high morbidity and mortality rates [1–6]. Despite their prevalence, IFD are still under-diagnosed in many countries, owing to a lack of knowledge among healthcare practitioners [7, 8] and the use of suboptimal diagnostic tools at healthcare facilities [9, 10]. Moreover, once identified, treatment of IFD remains particularly difficult [11] due to the scarcity of effective antifungal therapies [12, 13], along with associated toxicity [14] and constant monitoring requirements [15, 16], resulting in a huge unmet demand for patients and an increased healthcare burden.

Recently, the European Confederation of Medical Mycology (ECMM) released the results of a study done in 45 European countries to define the diagnostic methodologies used in clinical microbiology laboratories for IFD diagnosis and to examine the availability of antifungal therapies [17]. The study found significant differences between laboratories and countries, prompting national investigations to better understand specific concerns and support focused policy development [17].

We conducted a survey to determine the spectrum of diagnostic tests presently utilized by Italian institutions for IFD diagnosis and to define the current antifungal armamentarium available.

## Methods

This was a multi-centre questionnaire-based study that took place between March and November 2022, with data collected using an online electronic case report form hosted at www.clinicalsurveys.net/uc/IFI management capacity/. TIVIAN GmbH, Cologne, Germany (EFS Summer 2021). Each participant's answers were validated before the analysis to guarantee data correctness and completeness. The survey assessed variable topics relevant for diagnosis and treatment of IFD, including (a) institution profile, (b) perceptions on incidence and relevance of IFD at the particular institution, (c) microscopy, (d) culture and fungal identification, (e) serology, (f) antigen detection, (g) molecular assays, and (h) therapeutic drug monitoring (TDM). Participants were asked to respond dichotomously, stating whether or not the relevant method was accessible at their individual locations. In the case of serology, antigen detection, molecular testing, and TDM laboratories were approached to specify whether these methods were accessible onsite or at an outsourced institution. A Likert scale was used to estimate the prevalence of IFD, with responses ranging from 1 (extremely low) to 5 (very high) (Supplementary Table 1). Researchers from all regions of Italy, who are daily involved in the management of patients with IFIs (e.g., infectious disease specialists, microbiologists, internists), were contacted by mass email, which targeted not only the authors' close collaborators but also members of key scientific organizations such as the International Society of Human and Animal Mycology (ISHAM; www.isham.org), the European Confederation for Medical Mycology (ECMM; www.ecmm.info), [18] Epidemiological Surveillance of Infections in Haematological Diseases of Italy (SEIFEM; www. seifem.org), and the Italian Society of Anti-infective Therapy (SITA; www. sitaonline.net). To obtain the most accurate response, we screened online scientific repositories (ClinicalTrials.gov [19], EU Clinical Trials Register [20], Google Scholar [21], PubMed [19], and ScienceDirect [22]), as well as mycology publications. This approach was used to identify a broader pool of potential participants who are experts in the field of IFIs. In addition, online calls were launched on the social networks LinkedIn^®^ and Twitter^®^.

The participating institutions were classified according to their region's gross domestic product (GDP) per capita to evaluate whether there were any statistically significant variations in the availability of diagnostic tests or antifungals between Italian regions. A cut-off point was created, based on the Italian average GDP (29,661.5 € for year 2019) [23] dividing the country into regions with GDP of lower than 30,000 € and greater than 30,000 € (Supplementary Table 1). The data were summarized with frequencies and percentages. Proportions were presented in contingency tables and compared using Fisher’s exact test (variables with at least one cell with an expected value of 5) and the *X*^2^ test (variables with all cells having an expected value of > 5), where applicable. *P* values < 0.05 were deemed statistically significant. For statistical analysis, SPSS v27.0 was employed (SPSS, IBM Corp., Chicago, IL, United States).

## Results

**Fig. 1:**
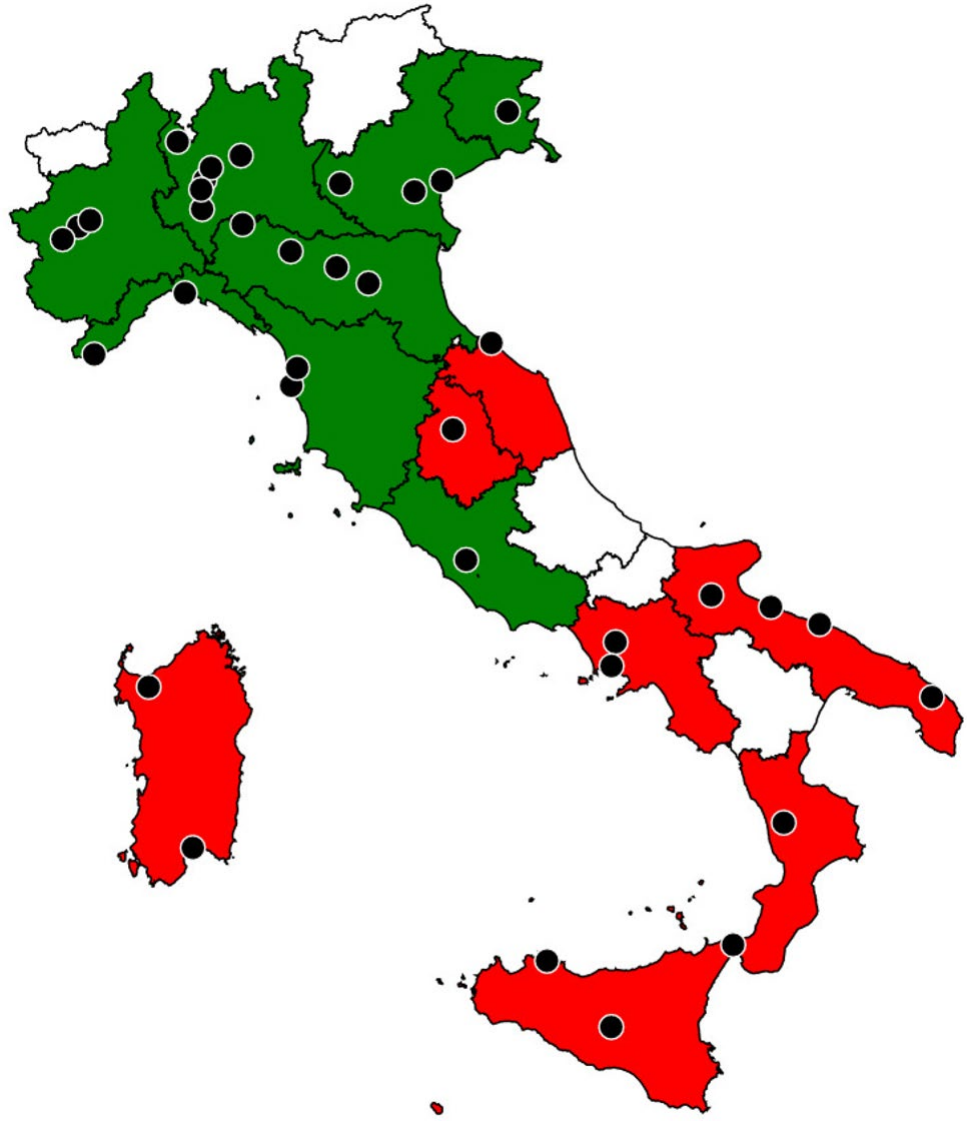
Map of participating institutions per region**.** Regions with a gross domestic product < 30,000 € are coloured in red. Regions with a gross domestic product > 30,000 € are coloured in green. Regions whose centres have not been included are coloured in white. If more than one participating centre from the same city, a single point is pictured

Throughout the study period, 49 researchers from 49 different institutions in 15/20 Italian regions responded to the online survey (Fig. 1). Among the responders, 26 (53.1%) were linked with university hospitals or national research institutions, 22 (44.9%) to non-university public hospitals, and one (2.0%) to a private hospital (Table 1). Patients with COVID-19 (*n* = 46, 93.9%), solid tumors (*n* = 43, 87.8%), haematological malignancies (*n* = 42, 85.7%), patients living with HIV or AIDS (*n* = 39, 79.6%), and patients who had undergone hematopoietic stem cell transplantations (*n* = 32, 65.3%) or solid organ transplantations (*n* = 29, 59.2%) were treated at the majority of institutions. Virtually all institutions (*n* = 43, 87.8%) were able to administer total parenteral nutrition, if required.

**Table 1:** Baseline characteristic of participating institutions in Italy

	Overall (*n* = 49)		GDP			
			< 30,000 € (*n* = 19)		> 30,000 € (*n* = 30)	
	*n*	%	*n*	%	*n*	%
Type of institution						
University hospital/National research institute	26	53.1	8	42.1	18	60.0
Public hospital	22	44.9	11	57.9	11	36.7
Private hospital	1	2.0	0	0.0	1	3.3
Target patients						
COVID-19	46	93.9	17	89.5	29	96.7
Diabetes mellitus	43	87.8	16	84.2	27	90.0
Parenteral nutrition	43	87.8	16	84.2	27	90.0
Oncology	43	87.8	15	78.9	28	93.3
Hematology	42	85.7	15	78.9	27	90.0
HIV/AIDS	39	79.6	14	73.7	25	83.3
Stem cell transplantation	32	65.3	9	47.4	23	76.7
Neonatal ICU	29	59.2	12	63.2	17	56.7
Solid organ transplantation	29	59.2	9	47.4	20	66.7
Access to onsite microbiology laboratory?	49	100.0	19	100.0	30	100.0
Mycological diagnostic procedures performed?						
Always in our institution	29	59.2	12	63.2	17	56.7
Part in our institution/part outsourced	20	40.8	7	36.8	13	43.3
IFD incidence						
Very low	2	4.1	2	10.5	0	0.0
Low	11	22.4	6	31.6	5	16.7
Moderate	25	51.0	11	57.9	14	46.7
High	11	22.4	0	0.0	11	36.7
Very high	0	0.0	0	0.0	0	0.0
Most important pathogen(s)						
* Aspergillus* spp.	45	91.8	17	89.5	28	93.3
* Candida* spp.	44	89.8	18	94.7	26	86.7
Mucorales	14	28.6	4	21.1	10	33.3
* Fusarium* spp.	10	20.4	2	10.5	8	26.7
* Cryptococcus* spp.	9	18.4	4	21.1	5	16.7
* Histoplasma* spp.	2	4.1	1	5.3	1	3.3

*COVID-19* coronavirus disease 2019, *€* Euros, *GDP* gross domestic product, *HIV/AIDS* human immunodeficiency virus/acquired immunodeficiency syndrome, *ICU* intensive care unit, *IFD* invasive fungal disease, *spp.* species

All Italian institutions reported having a microbiology laboratory, with 29 (59.2%) of them always performing mycological diagnoses on-site. Some tests were done on-site at the remaining 20 (40.8%) institutions, while others were outsourced to external laboratories (Table 1).

When considering epidemiology, a significant proportion of respondents classified the incidence of IFD at their institutions as either moderate (*n* = 25, 51.0%) or high (*n* = 11, 22.4%). The fungal pathogens most commonly causing concern were *Aspergillus* spp. (*n* = 45, 91.8%), *Candida* spp. (*n* = 44, 89.8%), *Mucorales* (*n* = 14, 28.6%), and *Fusarium* spp. (*n* = 10, 20.4%).

When questioned about microscopy performance (Table 2), 95% (*n* = 46) of the institutions reported having at least one or more microscopy methods available. The most accessible tools were China/India ink (*n* = 34, 69.4%), silver and Giemsa stains (*n* = 24, 49.0%, each), followed by potassium hydroxide (*n* = 15, 30.6%), and calcofluor white (*n* = 12, 24.5%). In terms of clinical suspicion of IFD, fewer than half of the Italian institutions (*n* = 21, 42.9%) always conducted direct microscopy of clinical specimens, 18.4% (*n* = 9) did so frequently, 24.5% (*n* = 12) sometimes, and 12.2% (*n* = 6) seldom. Only one institution stated that when an IFD was suspected, it never used direct microscopy.

**Table 2:** Comparison of available diagnostic techniques for mycological diagnosis in Italy

	Overall (*n* = 49)		GDP				
			< 30,000 € (*n* = 19)			> 30,000 € (*n* = 30)	
	*n*	%	*n*	%	*n*		%
Microscopy	46	93.9	17	89.5	29		96.7
Stains							
* China/India ink*	34	69.4	14	73.7	20		66.7
* Silver stain*	24	49.0	8	42.1	16		53.3
* Giemsa stain*	24	49.0	7	36.8	17		56.7
* Potassium hydroxide*	15	30.6	6	31.6	9		30.0
* Calcofluor white*	12	24.5	4	21.1	8		26.7
Direct microscopy frequency when IFD suspected							
* Never*	1	2.0	1	5.3	0		0.0
* Rarely*	6	12.2	4	21.1	2		6.7
* Sometimes*	12	24.5	6	31.6	6		20.0
* Often*	9	18.4	1	5.3	8		26.7
* Always*	21	42.9	7	36.8	14		46.7
Access to fluorescent dye	19	38.8	8	42.1	11		36.7
Direct examination in body fluids for suspected cryptococcosis	39	79.6	15	78.9	24		80.0
* India ink*	34	69.4	14	73.7	20		66.7
* Other dyes*	5	10.2	1	5.3	4		13.3
Direct microscopy for suspected mucormycosis	26	53.1	12	63.2	14		46.7
Silver stain for suspected pneumocystosis	21	42.9	8	42.1	13		43.3
Culture and fungal identification	49	100.0	19	100.0	30		100.0
Blood cultures for suspected fungemia	49	100.0	19	100.0	30		100.0
Fungal culture media							
* Sabouraud dextrose agar*	27	55.1	10	52.6	17		56.7
* Sabouraud dextrose agar* + *chloramphenicol*	26	53.1	11	57.9	15		50.0
* Sabouraud dextrose agar* + *gentamicin*	22	44.9	10	52.6	12		40.0
* Chromogen*	21	42.9	7	36.8	14		46.7
* Selective agar (chloramphenicol* + *cycloheximide)*	17	34.7	6	31.6	11		36.7
* Potato dextrose agar*	13	26.5	6	31.6	7		23.3
* Agar Niger*	10	20.4	6	31.6	4		13.3
* Lactrimel agar*	4	8.2	2	10.5	2		6.7
Available tests for specific identification	46	93.9	18	94.7	28		93.3
* MALDI–TOF–MS*	40	81.6	14	73.7	26		86.7
* Automated identification (i.e., VITEK*^*®*^*)*	33	67.3	14	73.7	19		63.3
* Biochemical tests (conventional mycology)*	28	57.1	13	68.4	15		50.0
* DNA sequencing*	26	53.1	8	42.1	18		60.0
* Mounting medium*	4	8.2	0	0.0	4		13.3
Antifungal susceptibility tests?	45	91.8	16	84.2	29		96.7
* For yeasts and moulds*	32	65.3	13	68.4	19		63.3
* For yeasts*	13	26.5	3	15.8	10		33.3
Available antifungal susceptibility test technologies							
* Broth microdilution, using EUCAST standards*	23	46.9	9	47.4	14		46.7
* VITEK*^®^	18	36.7	8	42.1	10		33.3
* E-test*^®^	18	36.7	5	26.3	13		43.3
* Broth microdilution, using CLSI standards*	18	36.7	3	15.8	15		50.0*
Maximum identification capability							
* Yeasts*	49	100.0	19	100.0	30		100.0
Genus/species	20	40.8	11	57.9	9		30.0
Genus/species/complex	19	38.8	4	21.1	15		50.0
Genus/species/complex/cryptic species	8	16.3	4	21.1	4		13.3
Genus	2	4.1	0	0.0	2		6.7
* Moulds*	49	100.0	19	100.0	30		100.0
Genus/species	41	83.7	17	89.5	24		80.0
Genus	8	16.3	2	10.5	6		20.0
Serology	41	83.7	16	84.2	25		83.3
* Aspergillus* spp.	39	79.6	16	84.2	23		76.7
* Onsite*	31	63.3	12	63.2	19		63.3
* Outsourced*	8	16.3	4	21.1	4		13.3
* Candida* spp.	28	57.1	13	68.4	15		50.0
* Onsite*	20	40.8	9	47.4	11		36.7
* Outsourced*	8	16.3	4	21.1	4		13.3
* Histoplasma* spp.	25	51.0	8	42.1	17		56.7
* Onsite*	14	28.6	4	21.1	10		33.3
* Outsourced*	11	22.4	4	21.1	7		23.3
Antigen detection	49	100.0	19	100.0	30		100.0
*Aspergillus* overall	42	85.7	17	89.5	25		83.3
* Aspergillus* galactomannan* ELISA*	47	95.9	19	100.0	28		93.3
Onsite	39	79.6	16	84.2	23		76.7
Outsourced	8	16.3	3	15.8	5		16.7
* Aspergillus* galactomannan* LFA*	18	36.7	8	42.1	10		33.3
Onsite	14	28.6	6	31.6	8		26.7
Outsourced	4	8.2	2	10.5	2		6.7
* Aspergillus LFD*	13	26.5	6	31.6	7		23.3
Onsite	10	20.4	4	21.1	6		20.0
Outsourced	3	6.1	2	10.5	1		3.3
1–3-Beta-d-glucan	42	85.7	14	73.7	28		93.3
Onsite	27	55.1	10	52.6	17		56.7
Outsourced	15	30.6	4	21.1	11		36.7
*Cryptococcus* overall	38	77.6	13	68.4	25		83.3
* Cryptococcus LAT*	38	77.6	15	78.9	23		76.7
Onsite	34	69.4	12	63.2	22		73.3
Outsourced	4	8.2	3	15.8	1		3.3
* Cryptococcus LFA*	20	40.8	9	47.4	11		36.7
Onsite	15	30.6	5	26.3	10		33.3
Outsourced	5	10.2	4	21.1	1		3.3
*Candida* antigen	22	44.9	11	57.9	11		36.7
Onsite	15	30.6	7	36.8	8		26.7
Outsourced	7	14.3	4	21.1	3		10.0
*Histoplasma*	18	36.7	7	36.8	11		36.7
Onsite	8	16.3	2	10.5	6		20.0
Outsourced	10	20.4	5	26.3	5		16.7
Molecular tests	41	83.7	16	84.2	25		83.3
*Pneumocystis* PCR	35	71.4	14	73.7	21		70.0
* Onsite*	29	59.2	11	57.9	18		60.0
* Outsourced*	6	12.2	3	15.8	3		10.0
*Aspergillus* PCR	32	65.3	12	63.2	20		66.7
* Onsite*	23	46.9	8	42.1	15		50.0
* Outsourced*	9	18.4	4	21.1	5		16.7
*Candida* PCR	25	51.0	9	47.4	16		53.3
* Onsite*	15	30.6	4	21.1	11		36.7
* Outsourced*	10	20.4	5	26.3	5		16.7
PCR for other fungi	19	38.8	8	42.1	11		36.7
* Onsite*	9	18.4	3	15.8	6		20.0
* Outsourced*	10	20.4	5	26.3	5		16.7
Mucorales PCR	18	36.7	9	47.4	9		30.0
* Onsite*	9	18.4	3	15.8	6		20.0
* Outsourced*	9	18.4	6	31.6	3		10.0

*This difference was considered as statistically significance, *p* = 0.032

*CLSI* Clinical and Laboratory Standards Institute, *DNA* deoxyribonucleic acid, *ELISA* enzyme-linked immunosorbent assay, *E test*^*®*^ epsilometer test, *EUCAST* European Committee on Antimicrobial Susceptibility Testing, *€* euros, *GDP* gross domestic product, *IFD* invasive fungal disease, *LAT* latex agglutination test, *LFA* lateral flow assay, *LFD* lateral flow device, *MALDI*—*TOF*, matrix-assisted laser desorption/ionization—time-of-flight mass spectrometer, *PCR* polymerase chain reaction, *spp*. species

All institutions had access to fungal culture medium and were able to run blood cultures to rule out fungemia. In 40 (81.6%) institutions, matrix-assisted laser desorption/ionisation time-of-flight spectrometry (MALDI-TOF-MS) was the most regularly utilized technology for species-level identification (Table 2). At 67.3% (*n* = 33) of the institutions, automated identification utilizing a VITEK^®^ system was available, whereas conventional biochemical testing was accessible in 57.1% (*n* = 28). Access to molecular methods was relatively limited, with just over half of the institutions (*n* = 26, 53.1%) offering deoxyribonucleic acid (DNA) sequencing.

Only yeast susceptibility testing was accessible in 26.5% (*n* = 13) of the laboratories, whereas testing for both yeast and moulds was available in 65.3% (*n* = 32). Broth microdilution according to the European Committee on Antimicrobial Susceptibility Testing Standards (EUCAST) was available in less than half of the institutions (*n* = 23, 46.9%), whereas access to broth microdilution according to the Clinical and Laboratory Standards Institute (CLSI) was reported in 36.7% (*n* = 18). Furthermore, 36.7% (*n* = 18) of the institutions employed the *E* test^®^ or VITEK^®^ automated system.

Serological testing was available at all participating facilities. The majority of institutions (*n* = 39, 79.6%) had access to *Aspergillus* spp. serology, followed by *Candida* spp. (*n* = 28, 57.1%) and *Histoplasma* spp. (*n* = 25, 51%). Serological testing was mostly available on-site, with roughly one-fifth of the institutions reporting serological antibody detection outsourcing.

The availability of antigen detection for several fungi, either on site or by outsourcing to other laboratories, was quite high in our survey, as indicated in Table 2. Antigen detection for *Aspergillus* spp. by galactomannan enzyme immunoassay was accessible at 47 (95.9%) institutions, with eight (16.3%) exclusively through an outsourced laboratory. Similarly, 42 (85.7%) institutions performed the test either on-site (*n* = 27, 55.1%) or through outsourcing (*n* = 15, 30.6%). The availability of *Cryptococcus* spp. latex testing was more limited, with 34 (69.4%) facilities performing the test domestically and 4 (8.2%) relying on an outsourced laboratory.

In 41 (83.7%) of the facilities, access to polymerase chain reaction (PCR) or other molecular tests was mentioned. Molecular testing targeted *Pneumocystis* spp. (*n* = 35, 71.4%), Aspergillus spp. (*n* = 32, 65.3%), or *Candida* spp. (*n* = 25, 51.0%) more often.

The availability of antifungal medication in Italy is shown in Table 3. Fluconazole and itraconazole (*n* = 45, 91.8% each) were the most often available triazoles, followed by voriconazole (*n* = 44, 89.4%). In 45 (91.8%) of the institutions, at least one echinocandin was accessible, mostly micafungin (*n* = 45; 91.8%). Flucytosine, terbinafine, and amphotericin B formulations (particularly liposomal formulation [*n* = 43, 87.8%] and lipid complex formulation [*n* = 15, 30.6%]) were available in 44 (89.8%), 24 (49.0%), and 7 (14.3%) facilities, respectively. When therapeutic drug monitoring (TDM) was evaluated, it was primarily accessible on-site for voriconazole (*n* = 23, 46.9%), while it was available in an outsourced laboratory in eight (16.3%) institutions. Posaconazole, itraconazole, and 5-flucytosine TDM were available at 22 (44.9%), 14 (28.6%), and 6 (12.2%) institutions, respectively, both on-site and in an outsourced laboratory.

**Table 3:** Comparison of available drugs for antifungal treatment in Italy

	Overall (*n* = 49)		GDP			
			< 30,000 € (*n* = 19)		> 30,000 € (*n* = 30)	
	*n*	%	*n*	%	*n*	%
Available antifungals						
* Amphotericin B*	44	89.8	18	94.7	26	86.7
Deoxycholate	9	18.4	1	5.3	8	26.7
Lipid complex	15	30.6	6	31.6	9	30.0
Liposomal	43	87.8	17	89.5	26	86.7
* Echinocandins*	45	91.8	18	94.7	27	90.0
Anidulafungin	36	73.5	15	78.9	21	70.0
Caspofungin	36	73.5	13	68.4	23	76.7
Micafungin	45	91.8	18	94.7	27	90.0
* Triazoles*	45	91.8	18	94.7	27	90.0
Fluconazole	45	91.8	18	94.7	27	90.0
Isavuconazole	39	79.6	16	84.2	23	76.7
Itraconazole	44	89.8	18	94.7	26	86.7
Posaconazole	41	83.7	18	94.7	23	76.7
Voriconazole	44	89.8	17	89.5	27	90.0
* Flucytosine*	24	49.0	11	57.9	13	43.3
* Terbinafine*	7	14.3	2	10.5	5	16.7
Therapeutic drug monitoring	31	63.3	7	36.8	24	80.0*
Flucytosine	6	12.2	1	5.3	5	16.7
* Onsite*	5	10.2	1	5.3	4	13.3
* Outsourced*	1	2.0	0	0.0	1	3.3
Itraconazole	14	28.6	3	15.8	11	36.7
* Onsite*	10	20.4	2	10.5	8	26.7
* Outsourced*	4	8.2	1	5.3	3	10.0
Posaconazole	22	44.9	3	15.8	19	63.3*
* Onsite*	16	32.7	2	10.5	14	46.7
* Outsourced*	6	12.2	1	5.3	5	16.7
Voriconazole	31	63.3	7	36.8	24	80.0**
* Onsite*	23	46.9	6	31.6	17	56.7
* Outsourced*	8	16.3	1	5.3	7	23.3

*GDP* gross domestic product, *US$* United States dollar

^*^This difference was considered as statistically significance, *p* = 0.005

^**^This difference was considered as statistically significance, *p* < 0.001

When the diagnostic capacity of participating institutions was compared by the GDP of their affiliated regions, only the use of broth microdilution using CLSI standards and therapeutic drug monitoring of antifungal agents revealed significant differences, with institutions located in regions with a GDP greater than 30,000 euros reporting more frequently (Tables 2 and 3).

## Discussion

This is Italy’s first nationwide assessment on the diagnostic and therapeutic capacities for patients with invasive fungal infections. The ECMM study group recently published a survey in 45 European nations, including Italy [17]. Nevertheless, limited numbers of Italian hospitals took part (*n* = 38), whereas the current study included 49 hospitals of various sizes and geographical regions. Our results, which provide a more comprehensive representation of the Italian condition at a national-level, show that our institutions seem well-prepared to manage IFD, with remarkably uniform adherence to current norms regardless of regional financing. Yet, the research indicated several diagnostic and treatment gaps, necessitating future attempts to improve IFD management and concentrate funding in relevant areas.

In terms of IFD epidemiology, one of the most significant factors to consider is the self-perception of the most relevant infections indicated by Italian respondents. According to the findings of this survey, *Aspergillus* spp. was the most important species, followed by *Candida* spp., Mucorales, and *Fusarium* spp. These findings contrast with the occurrence of infections caused by these pathogens in Italy in 2008 [24, 25], as well as recent European studies, which revealed a considerable concern, primarily for *Candida* spp. [17]. The enhanced awareness of *Aspergillus* spp. is probable due to the high number of patients with COVID-19 or haematological malignancies who are seen at participating institutions and are at a higher risk of acquiring invasive aspergillosis [26–28]. Yet, the rising worry in Italy regarding the development of azole-resistant *Aspergillus* spp. strains and the related high death rate may have contributed to the reported results [29–32].

This survey also suggests that, despite the presence of onsite microbiology laboratories in all Italian centres, approximately 40% of clinicians lacked complete access to all necessary tests within their own laboratories. This resulted in the need to send samples outside their hospitals for diagnostic testing. Consequently, concerns arise regarding the potential impact on turnaround times, particularly for centres managing complex patients, such as those with haematological malignancies or undergoing transplants.

Despite the fact that microscopy stains were accessible in more than 90% of centres, direct microscopy findings were always done on fewer than half of IFD suspects. This is in contrast to existing guidelines, which indicate that clinical specimens from patients suspected of having IFD should be analysed as soon as possible and that direct microscopy results should be reported as soon as possible to help in the diagnosis and management of IFD [33–35]. Moreover, our investigation discovered even poorer accessibility to direct testing employing optical brighteners, with calcofluor white staining available in less than 25% of the facilities studied. This conclusion cannot be attributable to cost concerns, as higher availability of this technology was reported in Asia/Pacific areas with similar GDP (60%) [17] and in Europe (~ 38%) in general [7, 9]. The restricted availability of this staining, which allows for the quick and presumptive identification of fungal species, might be addressed by advocating with key Italian institutions to increase the availability of calcofluor white stain, notably for the diagnosis of aspergillosis [35] and mucormycosis [12].

All of the Italian institutions surveyed were able to process materials for culture and fungal identification. Remarkably, more than 80% of respondents had access to the MALDI-TOF–MS technology for fast fungal identification, which is substantially higher than the proportion reported in Africa (17.5%) [36], Asia (43.0%) [17], and other European countries (~ 61.0%) [7, 9]. This high level of accessibility is good since MALDI-TOF-MS decreases response times [37], has proven to be cost-effective [37], does not require sophisticated technical expertise, and can definitely be useful for identifying *Candida* spp., *Aspergillus* spp., as well as rarer pathogenic species [38].

Regular antifungal susceptibility testing (AFST) is critical in the treatment of IFD patients. AFST not only assists in determining the most appropriate therapy for individual patients, but it also provides insight into local antifungal resistance patterns within healthcare facilities, which is critical in guiding the selection of optimal empirical treatment when an IFD is suspected [29]. Routine AFST is also useful for detecting inherent and recently acquired resistances [29]. According to our findings, most Italian laboratories can perform susceptibility testing for yeasts and moulds. Access to CLSI microdilution, on the other hand, was unevenly distributed, with only 15.8% of areas with GDP 30,000 euros having access to this approach, compared to 50% of regions with GDP > 30,000 euros (*p* = 0.001). In any case, the availability of EUCAST microdilution, Europe's preferred antifungal testing technique [39], was similar across Italian regions. The variance in CLSI microdilution availability might be attributed to places with lower economic resources investing in cost-effective and time-efficient commercial methods, such as *E* test^®^ or VITEK^®^, which have demonstrated a high correlation with standard procedures [29].

In terms of antifungal therapies, the primary antifungals were widely available and extensively dispersed throughout the country. The rate was approximately 90% not just for the World Health Organization (WHO)-defined essential medications [40], but also for additional pharmaceuticals such as isavuconazole (a first-line therapy together with voriconazole for invasive aspergillosis) or posaconazole (indicated for prophylaxis of fungal infections in high-risk haematological patients and for the treatment of mucormycosis). Certain systemic antifungals, such as 5-flucytosine and terbinafine, are likely unavailable due to low demand, as IFD needing these medications as first-line therapy is uncommon in Europe (e.g., disseminated cryptococcosis or scedosporiosis) [41–44].

Our research found numerous concerning impediments to TDM availability among participating universities. To begin, in comparison to recent European and Spanish surveys access to TDM in Italy was lower and generally inadequate, particularly for flucytosine (12.2%), itraconazole (28.6%), posaconazole (44.9%), and voriconazole (63.3%), for which routine dosing is now recommended by several guidelines [45]. Second, over half of the participating institutions sent blood samples to an outside laboratory for antifungal TDM, which might contribute to delays in findings and, eventually, dose modifications. Finally, there was an unequal distribution of TDM methodology throughout the country, with a substantial positive association between TDM availability and GDP. Given the potential benefits of TDM-guided dosage, such as enhanced therapeutic effectiveness [16, 46], reduced toxicity risk [15], cost savings, and decreased emergence of antifungal-resistant strains [47], addressing the limitations in TDM availability in Italy is deemed imperative. The limited accessibility to molecular tests observed in our survey underscores a further potential challenge that must be addressed in the next future.

The fact that the survey was done by email with a voluntary answer style that may result in an undetected response bias is the principal weakness of our study. In this regard, the majority of responses were from university hospitals or national research institutions, which often see a greater volume of patients and have more financial resources at their disposal. Thus, the results of this study may not be applicable to unspecialized clinical settings. Moreover, we did not collect data on Gram staining during the bacteriological processing of specimens. Although Gram stain is not specific to fungi, it theoretically has the potential to provide relevant clinical information that could impact the treatment approach for patients with suspected IFD. Lastly, it is crucial to note that our analysis was constrained by the impossibility to include centres from all areas of Italy. Yet, to the best of our knowledge, this survey is the broadest study ever undertaken to investigate the diagnostic capacities of invasive fungal infections in Italian laboratories.

In conclusion, our data show that overall diagnostic capacities for IFD in Italy are adequate, with no significant variations based on GDP except for TDM. Future efforts, in our opinion, should focus on enhancing the availability and usage of direct microscopic methods, as well as therapeutic medication monitoring, in order to achieve optimal treatment and improve patient outcomes.

### Supplementary Information

Below is the link to the electronic supplementary material.

Supplementary file1 (DOCX 31 KB)

## Data Availability

We ensure data availability for this manuscript, and all relevant data used in the research will be made accessible upon request to promote transparency and reproducibility.
